# Use of rubber dam versus cotton roll isolation on composite resin restorations’ survival in primary molars: 2-year results from a non-inferiority clinical trial

**DOI:** 10.1186/s12903-022-02449-y

**Published:** 2022-10-10

**Authors:** Isabel C. Olegário, Bruna L. P. Moro, Tamara K. Tedesco, Raiza D. Freitas, Ana Laura Pássaro, Jonathan Rafael Garbim, Rodolfo Oliveira, Fausto M. Mendes, CARDEC 03 collaborative group, Annelry Costa Serra, Antonio Carlos Lopes Silva, Carolina de Picoli Acosta, Caroline Mariano Laux, Cíntia Saori Saihara, Haline Cunha Medeiros Maia, Isabel Cristina Olegário, Isabella Ronqui de Almeida, Jhandira Daibelis Yampa Vargas, José Carlos P. Imparato, Julia Gomes Freitas, Karina Haibara De Natal, Kim Rud Ekstrand, Laura Regina Antunes Pontes, Mariana Bifulco, Mariana Minatel Braga, Mariana Pinheiro Araújo, Mayume Amorim do Vale, Renata Marques Samuel, Rita Baronti, Simone Cesar, Tathiane Larissa Lenzi, Tatiane Fernandes Novaes, Thais Gimenez, Cacia Signori, Maximiliano Sérgio Cenci, Daniela Prócida Raggio

**Affiliations:** 1 Division of Public and Child Dental Health, School of Dental Science, Trinity College Dublin, Dublin, Ireland; 2 Department of Paediatric Dentistry, School of Dentistry, University of São Paulo, São Paulo, Brazil; 3 School of Dentistry, Cruzeiro Do Sul University, São Paulo, Brazil; 4 School of Dentistry, University Dental Hospital (UDH), Cardiff University, Heath Park, Cardiff, CF14 4XW UK

**Keywords:** Rubber dam, Composite resin, Randomised clinical trial, Non-inferiority, Primary teeth, Children

## Abstract

**Background:**

This non-inferiority randomised clinical trial aimed to evaluate the survival of direct bulk fill composite resin restorations in primary molars using different methods of moisture control: rubber dam isolation (RDI—local anaesthesia and rubber dam) and cotton roll isolation (CRI—cotton roll and saliva ejector). Secondary outcomes included baseline and 2-year incremental cost, self-reported child’s pain scores and patient behaviour during the restorative procedure.

**Methods:**

A total of 174 molars (93 children) with dentine caries lesions were randomly allocated to study groups (RDI or CRI) and restored with bulk fill composite resin by trained operators. Two blinded examiners assessed the restorations for up to 24 months. Wong-baker faces and Frankl's behaviour rating scales were used for accessing the child's pain and behaviour, respectively. The primary outcome (restoration survival) was analysed using the two-sample non-inferiority test for survival data using Cox Regression (non-inferiority/alternative hypothesis HR > 0.85; CI = 90%). Bootstrap Linear regression was used for cost analysis and logistic regression for pain and behaviour analysis (α = 5%).

**Results:**

After 2-years, 157 restorations were evaluated (drop-out = 9.7%). The survival rate was RDI = 60.4% and CRI = 54.3%. The non-inferiority hypothesis was accepted by the Cox Regression analysis (HR = 1.33; 90% CI 0.88–1.99; *p* = 0.036). RDI was 53% more expensive when compared to the CRI group. No differences were found between the groups regarding pain (*p* = 0.073) and behaviour (*p* = 0.788).

**Conclusion:**

Cotton roll isolation proved to be non-inferior when compared to rubber dam for composite restorations longevity in primary molars. Furthermore, the latest presented the disadvantage of higher cost and longer procedure time.

*Clinical Significance* The moisture control method does not influence the longevity of composite restorations in primary molars. Cotton roll isolation proved to be non-inferior to rubber dam isolation and is a viable option for restoring primary molars.

*Clinical trial registration* registered NCT03733522 on 07/11/2018. The present trial was nested within another clinical trial, the CARies DEtection in Children (CARDEC-03-NCT03520309).

**Supplementary Information:**

The online version contains supplementary material available at 10.1186/s12903-022-02449-y.

## Background

The longevity of composite resin as a restorative material has already been demonstrated in the literature through systematic reviews [1–5]. Some authors claim that the contamination of the operative field can influence the bond strength and restoration longevity based on in vitro* studies* and indirect comparison in a systematic review [6–8]. However, there is a lack of clinical evidence to support this statement.

The most used isolation techniques for moisture control in dentistry involve using cotton rollers and saliva ejectors (cotton roll isolation—CRI) and dental clamps with a rubber dam (rubber dam isolation—RDI) [9]. Traditionally, using RDI is seen as an essential step towards clinical excellence in operative dentistry, especially related to composite materials [10]. In addition to having the potential to improve visibility and access to the operative field and to protect the patient from accidental swallowing or aspiration of dental instruments and materials, the use of a RDI aims to decrease the chances of contamination of the operative field. [11]

Laboratory studies show the harmful effects of salivary contamination on the bond strength of composite resin restorations, both to enamel and dentin [7, 12, 13]. In these studies, the decrease in bond strength after contamination by saliva or blood is mainly described. This happens because the saliva enzymes, mainly collagenases, can degrade exposed collagen fibres after acid etching, interfering in the hybrid layer formation. [9]

To reduce the procedure time, prevent salivary contamination during restorations, and facilitate the restorative technique, universal adhesives and bulk fill composite resins have been used in paediatric dentistry. The use of universal adhesives has already been clinically tested in primary dentition, showing no differences between self-etch and etch-and-rinse techniques [14]. Concerning the use of bulk fill composite in primary molars, there are few clinical trials with promising results on the survival of those restorations [15, 16]. However, the current recommendation and protocols used in those clinical trials involve RDI [6, 7].

Despite the advantages presented, rubber dam isolation in restorative procedures is often not used by professionals [17, 18]. The most common reasons for not performing this procedure can be due to low patient acceptance, longer consultation time and operator’s preference. When analysing the scientific evidence from randomised clinical trials [9, 19], results are highly heterogeneous and the studies presented a high risk of bias, especially regarding primary molars [4]. A systematic review that investigated the survival of adhesive restorations on primary teeth compared the isolation method used in the included studies, however, only an indirect comparison was made due to a lack of studies that directly compared the methods in their methodology [4].

Both for teaching practices and clinical decision-making, there is a need to investigate whether isolation is a factor that compromises the survival of composite resin restorations in primary teeth using well-designed randomised clinical trials. The present study aimed to evaluate the survival of direct bulk fill composite resin restorations in primary molars by comparing rubber dam isolation (RDI—local anaesthesia, use of dental clamp and rubber dam) and cotton roll isolation (CRI—cotton roll and saliva ejector).

## Material and methods

The report of the present paper followed the CONSORT (Consolidated Standards of Reporting Trials) guidelines.

### Trial design

This is a two-arm parallel single-blind non-inferiority randomised clinical trial. This study was registered on the Clinical Trials website under the registration number (NCT03733522).

The present trial was nested within another clinical trial, the CARies DEtection in Children (CARDEC-03-NCT03520309).

### Ethical considerations

The present trial was submitted and approved by the Ethics Committee of the University of Sao Paulo/Brazil (#3.065.654). Only children whose parents/legal guardians consented to participate in the study were considered for eligibility. The participant also needed to assent to take part on the research.

### Eligibility criteria

The inclusion criteria comprehended children aged between 4 to 8 years old, in good general health conditions and presented good behaviour in the initial consultation. Only children with at least one cavitated dentine caries lesion or failed restoration in a primary molar that required replacement and whose parents sought treatment at the University of São Paulo (clinics of paediatric dentistry) were consider eligible. The exclusion criteria included the presence of radiolucency into pulp or radiographic signs of pulp necrosis confirmed with a bitewing radiograph. Teeth that presented any clinical signs of severe pulp inflammation (spontaneous pain, nocturnal pain) or signs of pulp necrosis (clinical pulp exposure, pathological mobility, swelling or fistula) were excluded.

### Sample description

The sample size estimation was performed based on the primary outcome (restoration survival) based on a previous publication [20]. A survival rate of composite resin restorations after selective caries removal using rubber dam isolation of 66% was found after two years and used as a parameter for the sample estimation. A non-inferiority limit of 15% on the survival rate was considered (alternative hypothesis HR > 0.85). The sample size was increased by 40% for the cluster effect (more than one tooth could be included per child) and 10% to compensate for possible losses during the study. This gave a minimal sample size of 170 teeth. The sample unit was the tooth; more than one could have been included per child.

### Randomisation and allocation concealment

The children were randomly assigned into two groups: RDI and CRI. The randomisation process was generated by an external researcher who was not involved in the clinical procedures, using the website https://www.sealedenvelope.com/, and designed in blocks of different sizes (4, 6 and 8). Sealed, sequentially numbered, opaque envelopes were used and opened at the time of the restoration. A stratification of the randomisation list was performed considering the number of surfaces involved (single/multisurface) and restoration type (new restoration or restoration replacement). It was not possible to blind the operator and patient due to the clear differences in the protocols between study groups. Only the outcome assessors were blinded to groups.

### Operators and evaluators

All restorations and children’s treatment needs were performed by five trained dentists, including general practitioners (RF, JRG and RO) and specialists in paediatric dentistry (ICO and ALP). All operators had previous experience treating children and were previously trained in the provision of rubber dam isolation and restorative technique in children who were not included in this trial.

Two examiners (TKT and BLPM) were trained to conduct evaluations using Roeleveld et al. criteria [21]. Training consisted of examining restored primary teeth of children included in another clinical trial (CARDEC-03) and discussing the scores with a benchmark examiner (DPR) until an agreement was reached. Intra and inter-examiner calibration was performed before the trial and repeated early to ensure the examiners' agreement.

### Interventions

All primary molars were randomly allocated between the groups: Rubber dam isolation (RDI) and Cotton roll isolation (CRI) and restored with composite resin. In the RDI group, all teeth received previous local anaesthesia, and the rubber dam was placed aided by dental clamps). In the CRI group, no local anaesthesia was administered, and the isolation was performed only with cotton rolls and a saliva ejector.

Selective caries removal was performed in both groups (dentine-enamel junction was cleaned thoroughly while soft dentin layer was left in the cavity to avoid pulpal exposure). Caries removal was performed using high-speed round burs for cavity access (enamel removal), while DEJ was cleaned using a low-speed handpiece with a rose head bur or manual instruments (hand excavators appropriate to the cavity size). In the case of cavities involving proximal surfaces, a matrix and dental wedge were placed to reestablish the contact point. Scotchbond Universal Adhesive system (3 M ESPE) in a self-etch mode was applied using a microbrush and light cured for 10 s (Schuster Emitter B). Bulk fill composite resin (Filtek Bulk Fill composite resin—3 M ESPE) was inserted using a flat plastic composite spatula into the cavity in layers up to 4 mm and light cured for 30 s. Excess material was removed using finishing burs after checking contact points with articulation paper.

All information related to the patient (sex, caries experience-DMFT/dmft and child’s behaviour during the treatment) along with the clinical characteristics of the cavity (new restoration/replacement, number of surfaces involved: single/multisurface, jaw: upper/lower; molar: 1st or 2nd primary molar) were collected by the operators. An external researcher recorded the time spent in each restoration and all materials and instruments used during the procedure. The same researcher evaluated the pain reported by the patient at the end of the procedure.

### Evaluation of restorations

Two blind calibrated examiners (Kappa > 0.90) conducted the evaluations using Roeleveld et al. criteria [21] for up to 24 months. The scores 00 or 10 were considered a success, whilst scores 11, 12, 13, 20, 21, 30, 40 or 50 were considered a restoration failure. The remaining scores, 60, 70 and 90, were censored in the survival analysis. If a restoration failure was recorded, the dental team performed the replacement/repair of the restoration.

### Outcomes

The primary outcome of this trial is the restoration survival. As secondary outcomes, the differences between the groups' baseline and 2-year incremental cost were evaluated, as well as self-reported pain after treatment and the child's behaviour.

Additionally, the pain related to the dental treatment was assessed immediately after the treatment. The child was instructed by an external interviewer (who was not involved in the child’s treatment) to select the face that best reflected how they felt during treatment using the Wong-Baker Faces Pain Scale (WBFPS) [22]. The pain score was determined based on numerical values ranging from 1 to 6.

The child's behaviour was measured using Frankl's behaviour rating scale (FBRS) [23]. It consists of four behaviour categories ranging from definitely positive to definitely negative. The behaviour score was determined based on numerical values ranging from 1 to 4, and was assessed by the dentist (operator) at the end of the procedure.

### Estimation of costs

Costs for each group were estimated using a micro-costing approach, accounting for professional, instruments, and materials costs (payer's perspective). For this estimation, the operators registered the time spent, instruments, and materials used at each procedure using a specific form. An average price from three different Brazilian dental material supplies was used to determine the material costs, and quantities used during each procedure were registered. For the professional costs, we considered the minimum salary of a dentist and dental nurse according to the Brazilian Federal Law with a 40 h per week working regime (US$22.29/h and US$9.00/h, respectively). A life span of 3 years was accounted for instruments with a monthly usage of 160 h. All costs were calculated per molar in Brazilian Reais (R$) and converted to US Dollars (US$) using Purchasing Power Parities (PPP) currency values from 2020 [24] (1US$ = 2.311 R$).

### Statistical analysis

The analysis for the primary outcome (restoration survival) between groups was compared using the two-sample non-inferiority test for survival data using Cox Regression (non-inferiority/alternative hypothesis HR > 0.85; CI = 90%). Intention-to-treat analysis was conducted considering the proportion of treatment success at 2 years follow-up (using multiple imputations considering baseline variables) as a sensitivity analysis using non-inferiority test p-value and confidence interval (CI = 95%), derived from Miettinen and Nurminen's method [4]. These analyses were performed using NCSS Statistical software (NCSS 2021, USA).

As a secondary analysis, a shared frailty (child ID) Cox Regression analysis was performed to investigate the association of other independent variables (group, type of restoration, number of surfaces involved, 1st/2nd primary molars, caries experience and operator type) and restoration failure (two-tailed p values were reported). Treatment survival between groups was evaluated using Kaplan–Meier survival analysis and Log-rank test (α = 5%).

The baseline and 2-year incremental total cost between groups were compared using Linear regression analysis considering the child's level, and Bootstrap replications were set as 1000 using Stata 16.0 Software. As cost data presented non-parametric distribution initially, the linear model was built with a log-transformed dependent variable, and exponentiated coefficient was reported.

For the cost-effectiveness analysis (CEA), we considered the economic impact of using RDI instead of the CRI. The effect was the survival time of the restorations. Therefore, the differences between costs and effects of the strategies were calculated using the following equation:$\frac{\Delta Cost}{\Delta Effect}=\frac{Cost\, CRI-Cost\, RDI}{Survival \,CRI-Survival \,RDI.}$

A Bayesian approach was used to explore the uncertainties around the values obtained in the CEA. Firstly, data distribution was checked for cost and effects. Subsequently, a Monte-Carlo simulation (10′000) was conducted using XLSTAT 2020. The values were plotted into a cost-effectiveness plane (scatter plots). The proportion of points in each quadrant was calculated and assessed visually.

Children's self-reported pain and behaviour reported by the operator were compared between groups using ordinal logistic regression analysis considering the child's level (α = 5%).

## Results

**Fig. 1:**
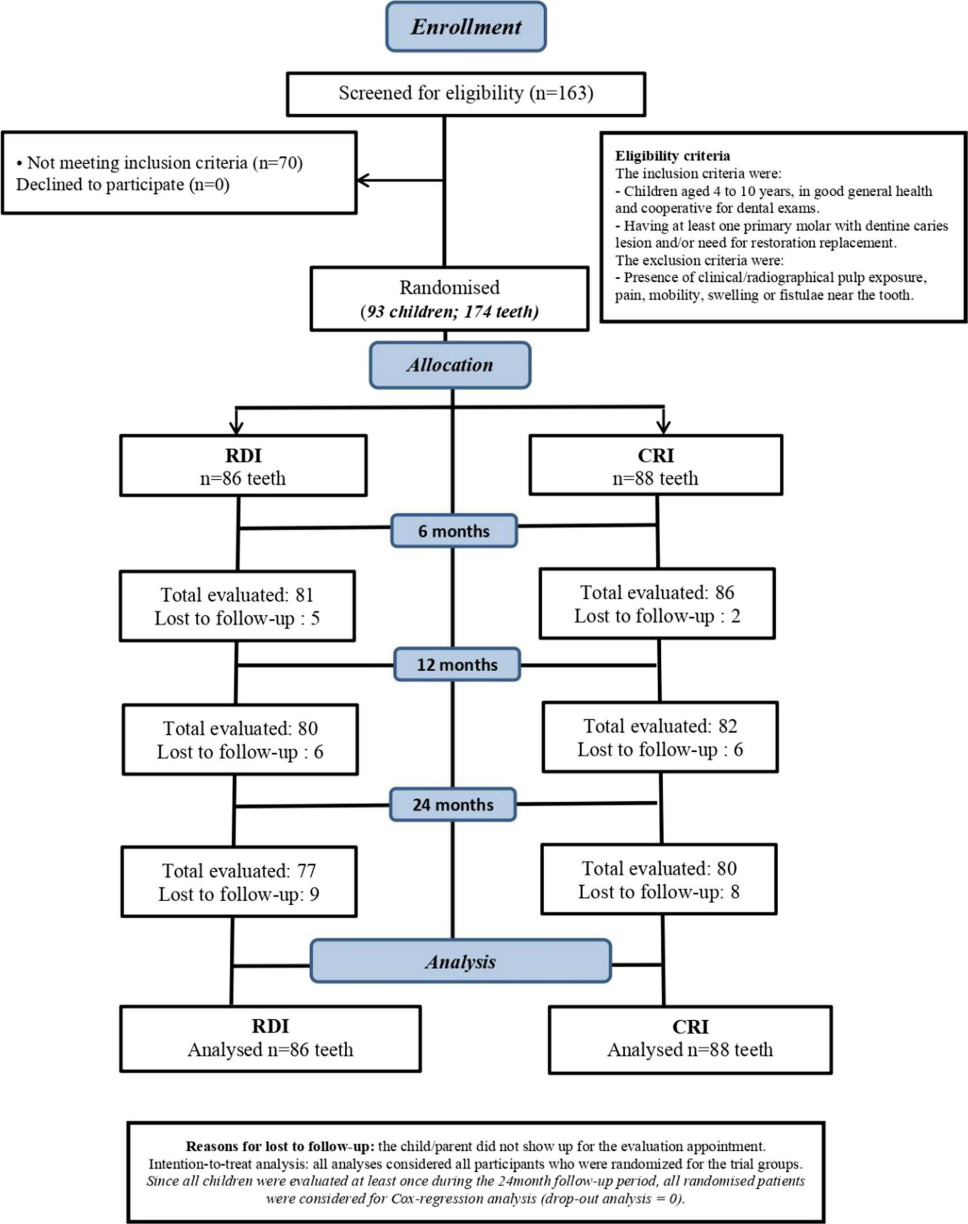
CONSORT Flow Diagram

Recruitment and treatment took place between December/2018 and March/2019. The follow-up started on June 5th, 2019, and lasted until March 20th/2021. The CONSORT flow diagram for clinical trials is presented in Fig. 1. After 2 years, 13 children (17 teeth) were not evaluated (drop-out = 9.77%). As all children were evaluated at least once during the evaluation period, they were included in the Cox regression analysis (Cox analysis drop-out = 0).

A total of 93 children were included in this study and received the interventions (n treated teeth = 174). Most participants were boys (55%), and the mean DMFT/dmft was 7.52 (± 3.61; min 1–max 16). The range of treated teeth per child was between 1 and 7 teeth. A total of 86 teeth were restored under RDI and 88 under CRI. Baseline demographic and clinical characteristics for each group and drop-out distribution are described in Table 1.

**Table 1:** Baseline characteristics between study groups and restoration drop-out after 24-months

	RDI	CRI	Stayed in	24-month Drop-out
Total N (%)	86 (49.43)	88 (50.57)	157 (90.23)	17 (9.77)*
Categorical variables—N (%)				
Sex				
Female	33 (49.25)	34 (50.75)	61 (91.04)	6 (8.96)
Male	53 (49.53)	54 (50.47)	96 (89.72)	11 (10.28)
Number of Surfaces				
Single surface (1)	23 (52.27)	21 (47.73)	38 (86.36)	6 (13.64)
Multisurface (> 1)	63 (48.46)	67 (51.54)	119 (91.54)	11 (8.46)
Molar				
First Molar	46 (50.00)	46 (50.00)	82 (89.13)	10 (10.87)
Second Molar	40 (48.78)	42 (51.22)	75 (91.46)	7 (8.54)
Operator				
Specialist	50 (52.63)	45 (47.37)	84 (88.42)	11 (11.58)
GDP	36 (45.57)	43 (54.43)	73 (92.41)	6 (7.59)
Restoration type				
New restoration	56 (50.45)	55 (49.55)	98 (88.29)	13 (11.71)
Restoration replacement	30 (47.62)	33 (52.38)	59 (93.65)	4 (6.35)
Continuous variable—mean (SD)				
DMFT/dmft	6.94 (3.62)	7.98 (3.73)	7.68 (3.42)	5.52 (5.44)
Number of surfaces	2.09 (1.02)	2.22 (1.05)	2.15 (1.01)	2.23 (1.34)

^*^9 children who dropped-out were from RDI group and 8 were from the CRI group (*p* = 0.760, by chi-square test)

**Fig. 2:**
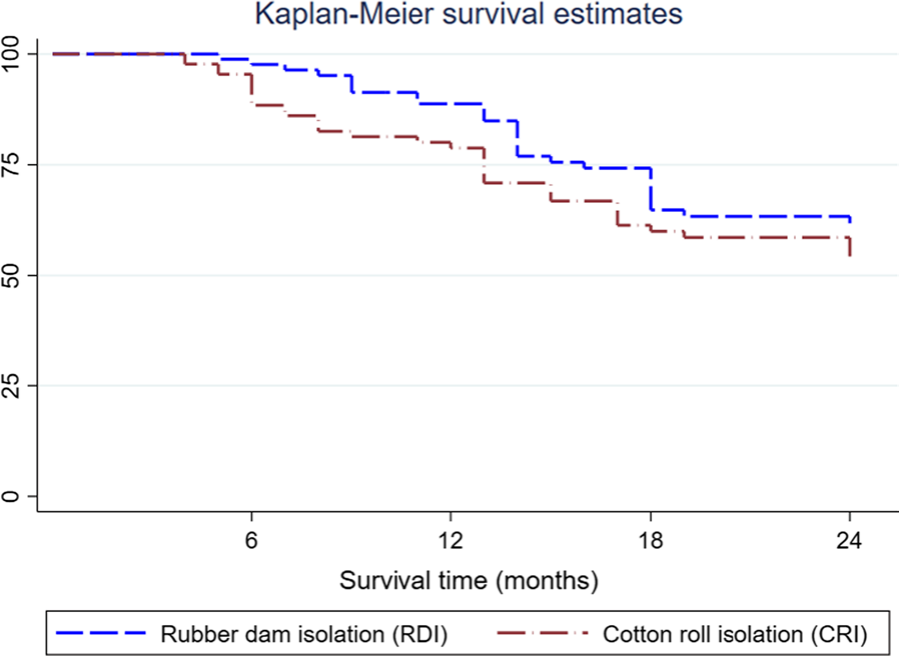
Kaplan–Meier Survival analysis between groups (log rank = 0.245)

**Fig. 3:**
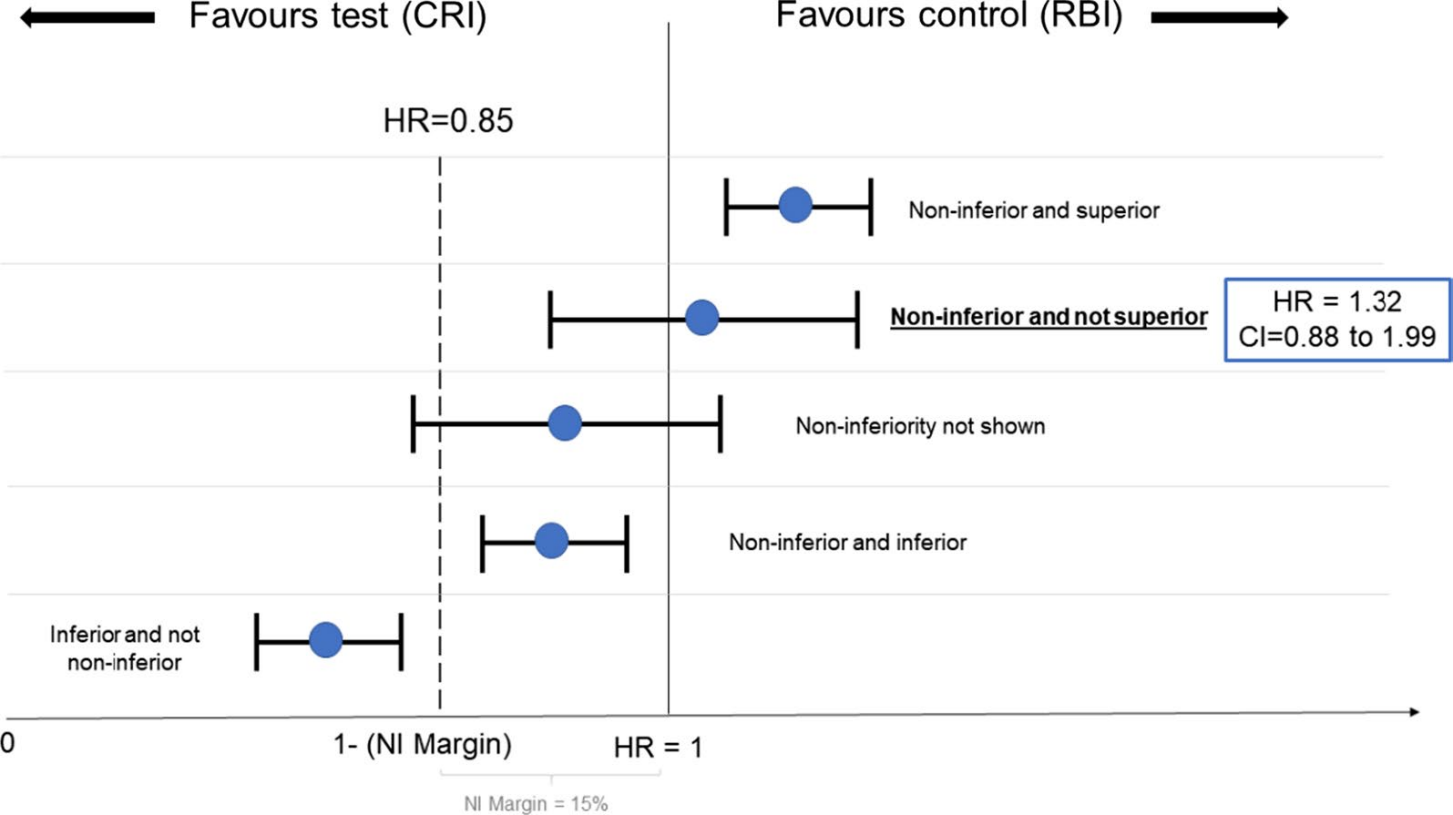
Possible results of a non-inferiority clinical trial considering a non-inferiority limit of 15% between groups using survival results as primary outcome (HR = 0.85)

The Kaplan–Meier survival plot is presented in Fig. 2. The survival rate after 2 years was RDI = 60.4% and CRI = 54.3% (log-rank *p* = 0.245). The primary outcome analysis using non-inferiority Cox regression and ITT analysis can be found in Table 2. The alternative non-inferiority hypothesis was accepted both by the Cox Regression analysis (HR = 1.33; 90% CI 0.88–1.99; *p* = 0.036) and Intention-to-treat analysis (success RDI = 62.79%; CRI = 57.95%; *p* = 0.003). An absolute difference of 5% was found between groups, and the lower confidence limit was − 9%. Since the non-inferiority limit of 15% was considered in this study, the non-inferiority between groups can be claimed. Figure 3 represents the possible results of non-inferiority clinical trials and a representation of the results found in the present research.

**Table 2:** Primary outcome analysis (restoration survival) using non-inferiority Cox Regression and Intention-to-treat analyses

Outcomes	RDI	CRI	*p* value
Primary outcome—Non-Inferiority Cox Regression analysis*			
% Survival	60.41%	54.31%	0.036
HR (90% C.L. of HR)	1.33 (0.88–1.99)		
Primary outcome—Intention-to-treat analysis (2 years) **			
N success/N total	54/86	51/88	0.003
% Success	62.79%	57.95%	
Absolute difference (95%CI)	0.05 (− 0.09 to 0.19)		
OR (95%CI) **	1.22 (0.67–2.25)		0.201

HR = Hazard Ratio; OR = Odds ratio

Ha = non-inferiority at α = 5%

^*^ 100(1−2α)% Confidence Interval and p-value for non-inferiority survival data (Wald test)

^**^ p values and 95% CI were derived by Miettinen and Nurminen’s method using non-inferiority test for two proportions

The Cox regression analysis of prognostic factors to the restoration failure is presented in Table 3. No difference was found between the restoration failure and independent variables in the univariate and adjusted analysis. The reason for restorations failure was mainly related to the bulk-fracture of the restoration (score 30; 52.24%), followed by secondary caries within dentine (score 21; 25.37%) and defect at the margin > 0.5 mm in depth (score 11; 4.48%). Only 2 restorations presented with inflammation of the pulp where extraction or pulpectomy was required (score 40; 2.99%). This was due to a previously failed restoration (RDI = 1; CRI = 1) that led to plaque accumulation and caries progression.

**Table 3:** Univariate and adjusted two-tailed Cox Regression Analysis between restorative treatment failure and prognostic factors

Variable	Survival rate %	95% CI	HR Univariate 95% CI	*p*-value	HR Adjusted 95% CI	Two-tailed p-value
Group						
RDI (ref)	60.41	48.40–70.47	1.36 (0.82–2.23)	0.222	1.34 (0.81–2.19)	0.244
CRI	54.31	42.52–64.67				
Restoration						
New restoration (ref)	63.16	52.83–71.83	1.54 (0.95–2.52)	0.079	1.53 (0.93–2.52)	0.093
Replacement	46.43	32.60–59.16				
Number of Surfaces						
Single (ref)	62.65	45.69–75.64	1.18 (0.66–2.11)	0.565	1.08 (0.60–1.95)	0.782
Multiple	55.43	45.74–64.08				
Molar						
1st molar (ref)	52.95	41.36–63.25	0.72 (0.44–1.18)	0.203	–	–
2nd molar	62.00	49.79–77.05				
Caries experience (DMFT/dmft)						
Low (1–3) (ref)	52.38	26.54–72.97	1.02 (0.48–2.16)	0.956	–	–
High (> 3)	57.67	48.86–65.51				
Operator						
Specialist (ref)	56.70	45.27–66.61	1.04 (0.63–1.69)	0.872	–	–
GDP	58.20	45.75–68.75				
TOTAL	57.30	49.01–64.74				

HR = Hazard ratio; CI = Confidence Interval; SE = Standard Error *p* < 0.05–95% CI

Adjusted analysis considered only study group, type of restoration and number of surfaces

**Fig. 4:**
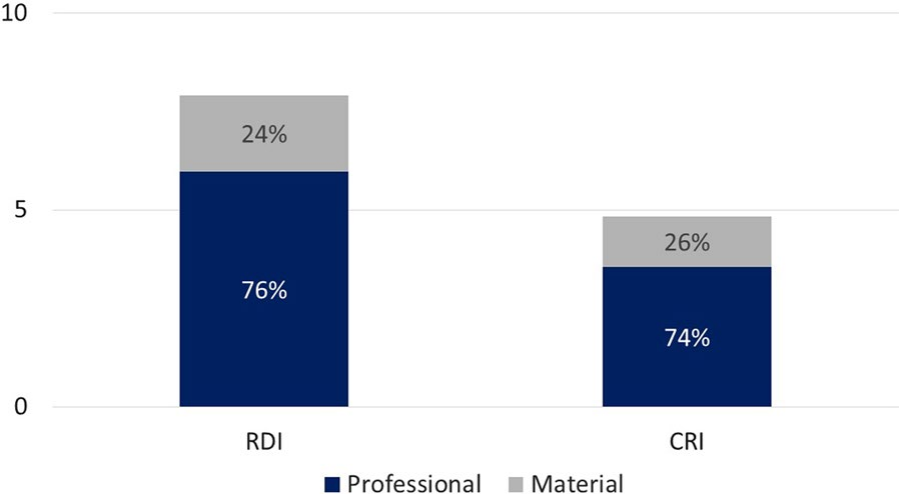
Distribution between mean professional and material baseline cost between study groups in US$

The mean (SD) time in minutes spent in RDI and CRI were 30.19 (SD = 12.47) and 17.85 (SD = 10.06), respectively. Regarding restoration costs, the professional component was the most expressive proportion, representing more than 74% of the treatment cost in the baseline (Fig. 4). At baseline, RDI was the most expensive option, requiring an investment of US$ 17.65 per restoration (Table 4). When considering the incremental cost (replacement/repairs) after failures during the 2-years follow-up, RDI was 53% more expensive when compared to the CRI group (2-year cost; RDI = US$24.62; CRI = US$16.11). Moreover, multiple surfaces restoration presented higher costs at baseline and after 2 years (*p* < 0.001).

**Table 4:** Evaluation of the baseline and 2-year incremental cost between groups and number of surfaces over time using Bootstrap regression analysis (1000 repeats) using Linear Regression considering the child level

Cost analysis	Mean US$ (SD)	Univariate analysis		Adjusted analysis	
		Coefficient (SE) 95% CI	*p* value	Coefficient (SE) 95% CI	*p* value
Baseline Total Cost					
Study Groups					
RDI (ref)	17.65 (6.62)	− 6.88 (0.89)	< 0.001*	− 6.95 (0.93)	< 0.001*
CRI	10.76 (5.09)	− 8.64 to − 5.12		− 8.79 to − 5.11	
Number of surfaces					
Single (ref)	12.65 (5.97)	2.01 (1.04)	0.053	2.28 (0.76)	0.003*
Multiple	14.67 (7.03)	-0.02 to 4.06		0.77–3.78	
2 years Total Cost					
Study Groups					
RDI (ref)	24.62 (19.42)	− 8.51 (2.44)	< 0.001*	-8.69 (2.45)	< 0.001*
CRI	16.11 (10.64)	− 13.30 (− 3.72)		− 13.49 to − 3.88	
Number of surfaces					
Single (ref)	15.95 (7.25)	5.83 (1.89)	0.002*	6.16 (1.94)	0.001*
Multiple	21.79 (17.98)	2.11–9.56		2.36 to 9.97	

CI = Confidence interval; SE = Bootstrap Standard error; SD = standard deviation; *p < 0.05

All costs were measured in Brazilian reais (R$) and converted to US Dollars (US$) using purchasing power parities (PPP)– Conversion rate 1US$ = 2.311R$

**Fig. 5:**
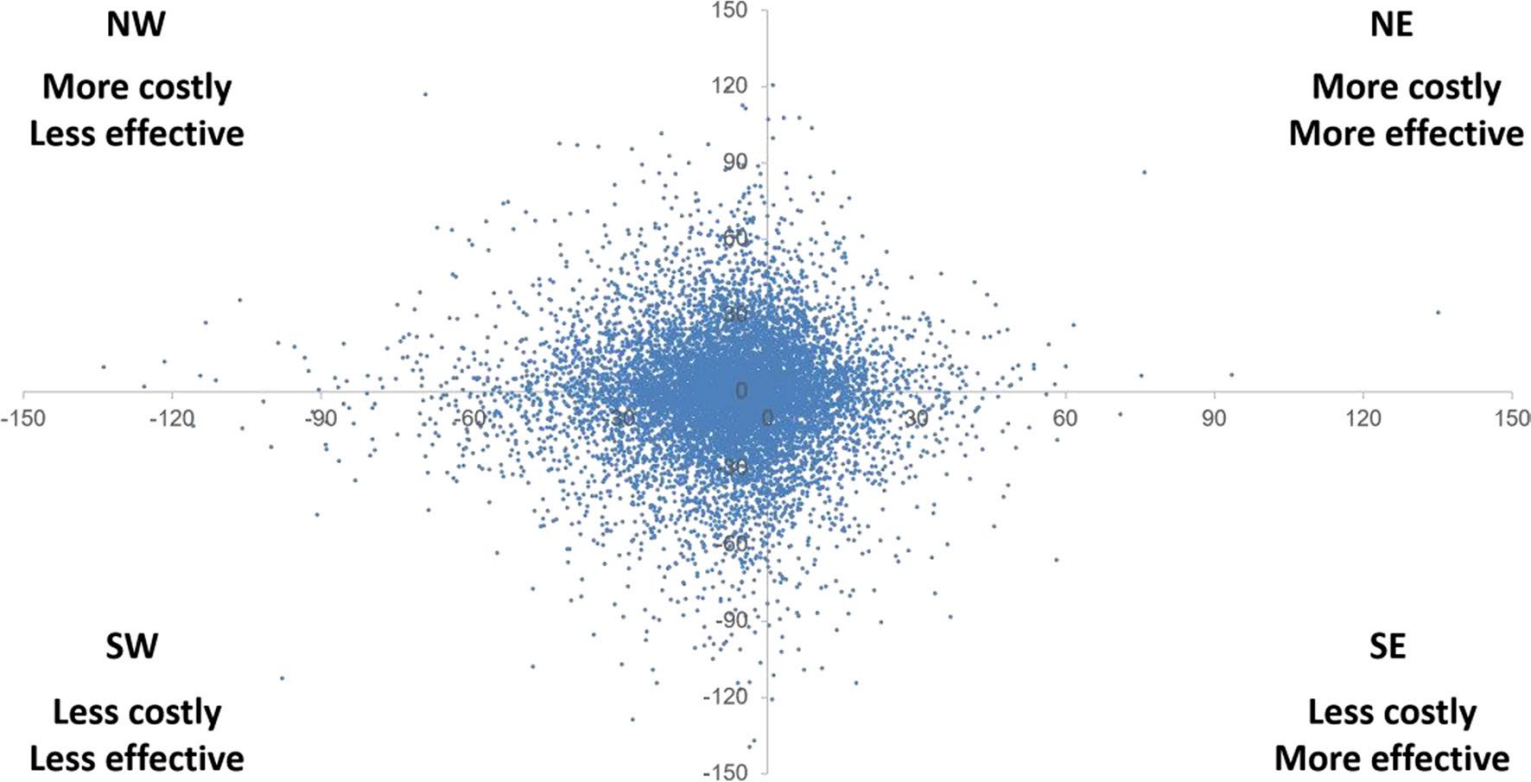
Cost-effectiveness of using CRI versus RDI considering costs (US$) and effectiveness (survival in months)

There is considerable uncertainty surrounding the cost-effectiveness of the interventions. Probabilistic sensitivity analysis indicates a 51% probability for the CRI to be less costly than the RDI (South-East and South-West quadrants). When considering the survival of the restorations in months, there is a 30% probability for the CRI to be cost-effective (Northeast and Southeast quadrants) compared with the RDI, considering a maximum willingness-to-pay of approximately US$120 (Fig. 5).

**Fig. 6:**
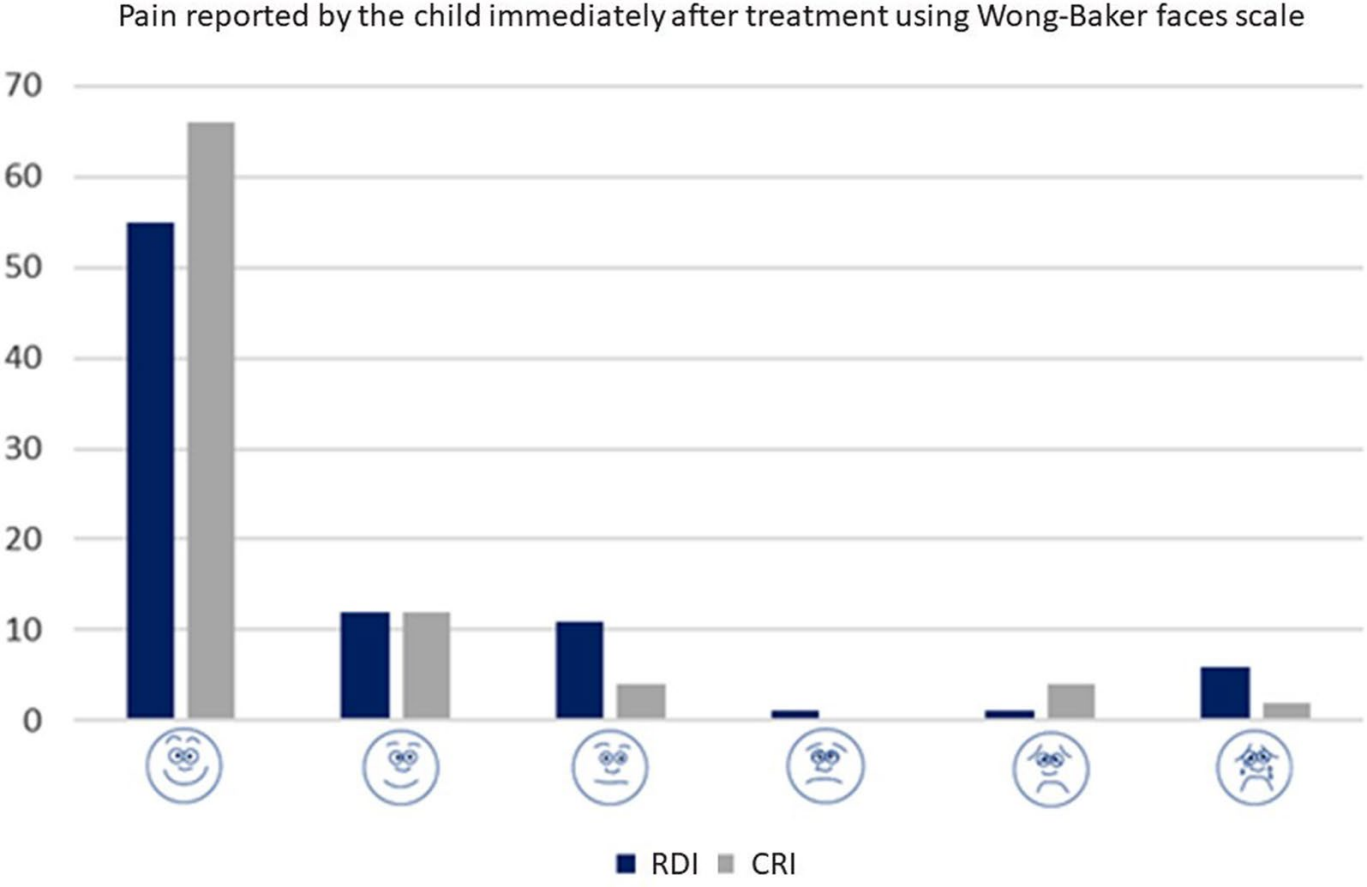
Distribution between pain reported by the child after treatment between groups

**Fig. 7:**
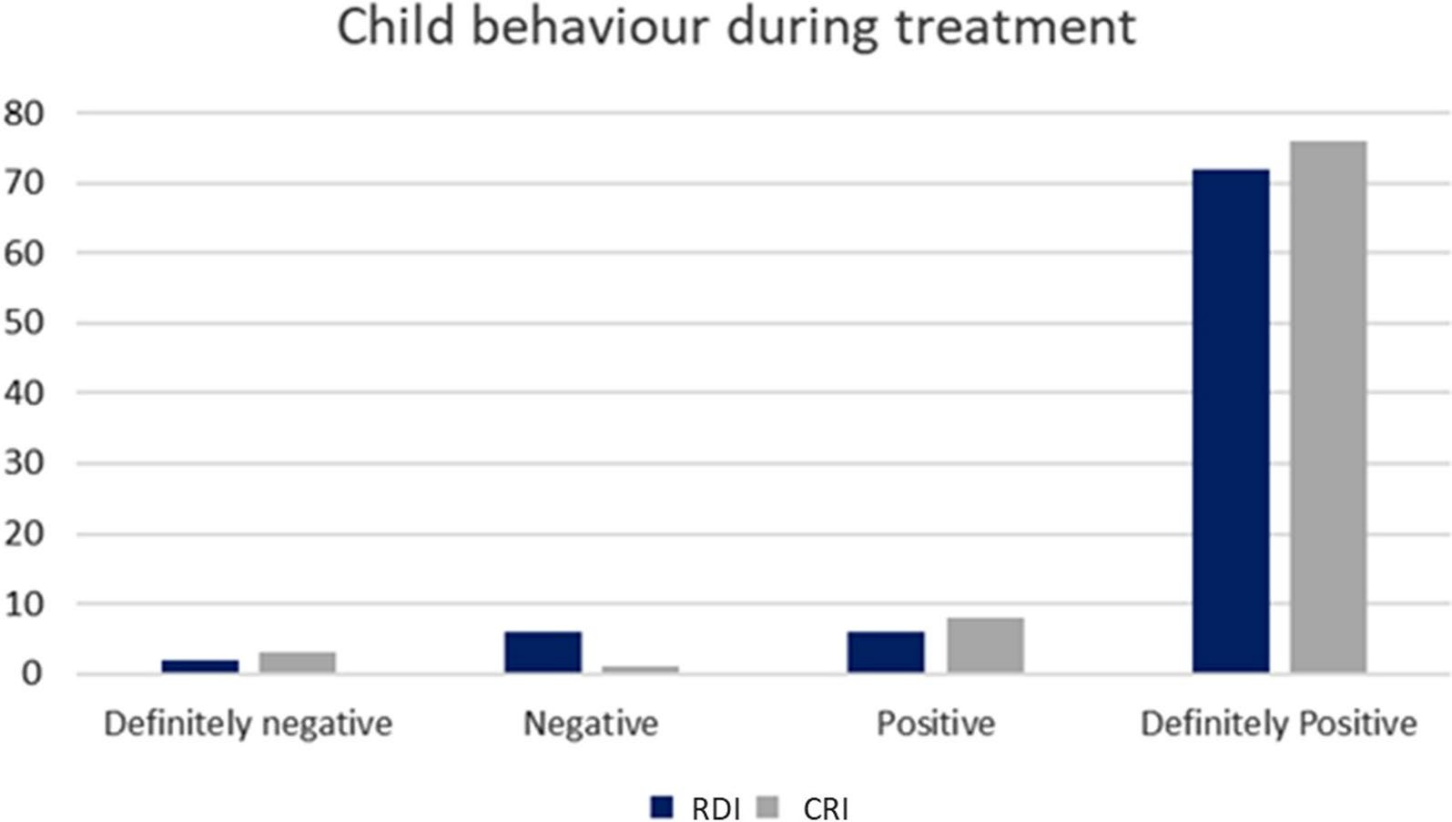
Distribution between child behaviour (Frankl's behaviour rating scale—FBRS) reported by the operator after treatment between groups

No differences were found between the groups in pain reported by the child using Wong-Baker faces scale using ordinal logistic regression considering the child cluster (OR = 0.58; CI = 0.32–1.05; *p* = 0.073). Figure 6 shows the distributions of the facial scores between study groups. Similarly, no differences were found between the children's behaviour during the restorative treatments (Fig. 7), and the majority of children presented ‘definitely positive’ behaviour according to Frankl’s behaviour rating scale (OR = 1.14; 95%CI = 0.43–2.97; *p* = 0.788).

## Discussion

This randomised clinical trial compared the survival between composite restorations in primary molars using two different methods of tooth isolation: RDI and CRI. A systematic review [25] that investigated the use of RDI compared to other forms of tooth isolation for both primary and permanent teeth found no robust evidence to favour rubber dam usage. Two studies were included for primary teeth restorations, but none investigated the use of composite resin. Due to the low quality of evidence, more randomised controlled trials with longer follow‐up were suggested to investigate if the type of isolation could influence the restoration success.

Our hypothesis was that the survival rate of composite restorations using cotton roll isolation (CRI) is non-inferior (not worse) than the use of rubber dam isolation (RDI). For that reason, a non-inferiority trial design was chosen, and a non-inferiority margin of 15% was set as the maximum difference between the treatments for which the outcomes can be considered "non-inferior". The lower confidence interval limit of Cox regression analysis (HR) and intention-to-treat analysis (OR) was greater than 0.85. Our non-inferiority limit was set as 15%, so we can affirm that restorations using CRI are non-inferior to RDI. Moreover, looking at the CI upper bond results, we can also affirm that CRI is not superior to RDI (Fig. 3). Therefore, future recommendations for composite restorations in primary teeth should also consider using cotton roll isolation as an alternative to rubber dam.

The survival rate for composite resins in the present study was 60.41% for RDI and 54.31% for CRI after 2 years. This result is close to the estimated survival of 66% used for sample size calculation [20]. If a restoration failure was detected, it was considered a failure for the primary outcome evaluation; however, all failed restorations receive appropriate treatment depending on the extension of the defect (restoration repair or replacement of the restoration). After 24 months, only 2 restorations presented inflammation at the pulp level, which required endodontic treatment or extraction. Therefore, although this study reported an annual failure rate (AFR) of 21%, the tooth survival (percentage of teeth that remained without pulp inflammation—pain/symptoms free) at the end was 98.8%.

The restoration's survival did not differ between the operators (specialists in paediatric dentists and general dental practitioners). This could be explained as all operators received training in handling the materials before the start of the trial and were also experienced in treating paediatric patients as part of the CARDEC trials clinic. Another factor that has been reported to influence the survival of the restorations is the number of surfaces involved [26]. Occlusal restorations tend to present a higher survival when compared to occlusoproximal restorations in primary teeth [4, 5]. In the present trial, no difference was found between single and multiple surface lesions (HR = 1.08; CI = 0.60–1.95). However, single surface restorations in the inclusion criteria were not limited to occlusal surfaces. We have also included buccal, palatal and mesial surfaces, which could have contributed to a similar failure rate to multisurface restorations.

The present trial also looked at the cost of the restorations as a secondary outcome [27]. As expected, the cost of using RDI was higher compared to CRI in both baseline and after two years, considering the incremental cost. The higher cost of the procedure was influenced both by the professional cost (due to the time spent in each restoration) and by the number of materials needed to perform a rubber dam isolation compared to CRI.

The CEA demonstrated more than 50% probability for the CRI to be less costly than the RDI, considering both more and less effectiveness. However, when considering gains in health effects, the probability of the CRI being cost-effective is 30%, depending on the willingness to pay. Some methodological aspects of our economic evaluation should be highlighted. This analysis was performed based on a Brazilian context (both in terms of income and material cost) and should be interpreted with caution if extrapolating to other countries.

The time horizon was the study’s follow-up, and a wider time frame would allow a better understanding of the long-term effects. Moreover, the cost-effectiveness of the CRI depends on the willingness-to-pay threshold, which depends on the opportunity costs where the strategy will be implemented, and this was not on the scope of the present evaluation. Thus, from our results, it is not possible to make a strong recommendation for the implementation of the strategy in a healthcare system; however, this is the first study demonstrating that the CRI may be associated not only with better health outcomes but also with lower costs when compared to the RDI.

As important as looking at the survival and cost of the restoration, it is to investigate patient-reported outcomes (PROs) [28]. The present trial investigated the child’s pain reported by the child immediately after treatment using the Wong Baker faces scale. The use of RDI requires local anaesthesia for the placement of a dental clamp. We expected that children who underwent local anaesthesia for restorative treatment could influence the pain scores. However, no difference was found between the groups; most children reported positive responses (happiest face). Moreover, the analysis of the child's behaviour during the treatment reported by the operator (using Frankl's behaviour scale) also did not show a difference between the groups. This could be explained first by the characteristics of the study population. Those children who sought dental treatment in our clinic had a low socioeconomic background and good behaviour, and their parents were very pleased to receive the free of charge dental care. Moreover, the operators were experienced in treating children and applying behaviour management techniques (such as tell-show-do and positive reinforcement) [29] throughout the treatments, which may have helped provide this positive restorative experience in both groups. Another factor that could have influenced the pain scores is the age of the participants. There is still no robust evidence when it comes to the evaluation of psychometric properties, especially for measuring self-report pain in children younger than 6 years [30]. Although only 11 participants were under the age of 6 years (11.82% of the sample) and only two participants were 2-years-old, this could be a source of bias for the secondary outcome of the present trial.

In conclusion, cotton roll isolation proved to be non-inferior compared to rubber dam isolation for composite restorations in primary molars in restoration survival. No difference was found concerning the pain reported by the child and the child's behaviour assessed by the operator. Therefore, for both teaching practices and clinical decision-making, cotton roll isolation can be recommended as well as rubber dam isolation for composite restorations; however, the latest present the disadvantages of higher cost and longer procedure time.

## Conclusion

Cotton roll isolation proved to be non-inferior in terms of restoration longevity when compared to rubber dam isolation and, therefore, can be used as an alternative technique for moisture control in the provision of composite restorations in primary molars. Furthermore, the latest presented the disadvantage of higher cost and longer procedure time.

## Supplementary Information

**Additional file 1.** Survival analysis isolation methods - raw data.

## Data Availability

All data generated or analysed during this study are included in this published article and its Additional file 1.

## Abbreviations

RDI: Rubber dam isolation
CRI: Cotton roll isolation
WBFPS: Wong-baker faces pain scale
CONSORT: Consolidated standards of reporting trials
FBRS: Frankl’s behaviour rating scale
R$: Brazilian Reais
PPP: Purchasing power parities
CEA: Cost-effectiveness analysis
CI: Confidence interval
AFR: Annual failure rate
HR: Cox regression analysis
PROs: Patient reported outcomes
